# System modeling reveals the molecular mechanisms of HSC cell cycle alteration mediated by *Maff* and *Egr3* under leukemia

**DOI:** 10.1186/s12918-017-0467-4

**Published:** 2017-10-03

**Authors:** Rudong Li, Yin Wang, Hui Cheng, Gang Liu, Tao Cheng, Yunlong Liu, Lei Liu

**Affiliations:** 1 Center for Computational Biology and Bioinformatics, Department of Medical and Molecular Genetics, Indiana University School of Medicine, Indianapolis, IN 46202 USA; 2 Shanghai Public Health Clinical Center and Institutes of Biomedical Sciences, Fudan University, Shanghai, 200031 China; 3 Institute of Hematology, Chinese Academy of Medical Sciences and Peking Union Medical College, Tianjin, 300020 China; 4 Shanghai Center for Bioinformatics Technology, Shanghai, 201203 China

**Keywords:** System modeling, Hematopoietic stem cells, *Maff* and *Egr3*, Leukemia, Model selection

## Abstract

**Background:**

Molecular mechanisms of the functional alteration of hematopoietic stem cells (HSCs) in leukemic environment attract intensive research interests. As known in previous researches, *Maff* and *Egr3* are two important genes having opposite functions on cell cycle; however, they are both highly expressed in HSCs under leukemia. Hence, exploring the molecular mechanisms of how the genes act on cell cycle will help revealing the functional alteration of HSCs.

**Results:**

We herein utilize the bioinformatic resources to computationally model the acting mechanisms of *Maff* and *Egr3* on cell cycle. Using the data of functional experiments as reference, molecular acting mechanisms are optimally enumerated through model selection. The results are consolidated by evidences from gene sequence analysis, thus having enhanced the confidence of our pilot findings, which suggest that HSCs possibly undergo a “adaptation - suppression” process in response to the malignant environment of leukemia.

**Conclusion:**

As a pilot research, our results may provide valuable insights for further experimental studies. Meanwhile, our research method combining computational modeling and data from functional experiments can be worthwhile for knowledge discovery; and it can be generalized and extended to other biological/biomedical studies.

**Electronic supplementary material:**

The online version of this article (doi:10.1186/s12918-017-0467-4) contains supplementary material, which is available to authorized users.

## Background

*Maff* and *Egr3* are two important regulatory factors in hematopoiesis development. Previous studies showed that *Maff* was mainly responsible for the transcription regulation of megakaryote differentiation (towards platelet) [1–3]; and less was known for the functions of *Egr3* in cell cycle [4]. Both Maff and Egr3 are able to recognize certain DNA elements, thus enhancing the transcriptions of their target genes [5, 6]. We had demonstrated via gene over-expression and in vitro cell culture in our previous study that *Maff* stimulated cell cycle (i.e. pro-proliferation); and *Egr3* potently suppressed cell cycle (i.e. counter-proliferation) [7, 8]. In addition, we found via molecular profiling that *Egr3* up-regulated *p18* and *p19*, two cyclin-dependent kinase inhibitors (CKIs), which might be how *Egr3* suppressed cell cycle [7]. However, we also discovered that both *Maff* and *Egr3* were highly expressed in hematopoietic stem cells (HSCs) under leukemia (especially at the late stage, i.e. ≥ day 14), in which the cell cycles of most HSCs were heavily suppressed [7–9]. Therefore, the molecular mechanisms of how *Maff* and *Egr3* act on cell cycle and why the two functionally-opposite genes are both highly expressed under leukemia, remain to be revealed.

Nonetheless, forthcoming experiments with respect to molecular regulations are preceded by a major concern that there are usually too many molecular interactions, outnumbering the experimental capacity. Hence direct experimentation is possibly not affordable. Meanwhile, simplistic a priori computational methods such as hyperpositioning of static molecular networks, as in most previous researches, cannot adequately solve the question. As it is known, cell cycle is governed by the dynamic states of cyclins and cyclin-dependent kinases (CDKs), as well as various (positive/negative) regulatory factors. On the other hand, cell cycle dynamics of many prokaryotic/eukaryotic organisms are well-understood and mathematical models are established [10]. Therefore, we herein conduct a pilot study, using mathematical modeling and data of prior experiments to refine/optimize the possible ways of molecular actions. First, we use information of molecular interactions to indicate possible actions related to *Maff*/*Egr3*. Second, we numerically test the possibilities with experimental data of HSC cell cycle [7, 8]. The tests are implemented on a curated cell cycle dynamic model with organism comparability to our experimental data [11–13], aiming to find out the ways of actions giving rise to the particular kinetic phenomena observed in the experiments. And finally, optimal possibilities that pass the tests, i.e. the ways of actions that should be in place so that the experimental phenomena can be observed/explained, are enumerated.

We have computationally yielded a (minimal) set of molecular actions that can explain the particular cell cycle kinetics and gene expression profiles of HSCs under leukemia. Our results implicate on the molecular level that HSCs tend to be cancerized by the stress of leukemia, but eventually they suppress the functionality as self-protection. The results may provide pilot knowledge and additional insights to further studies of leukemia-induced functional alterations of hematopoietic cells. Meanwhile, our computational modeling method can be generalized and serve for other biological or biomedical studies.

## Results

### Gene expressions showed significantly differential profiles of *Maff* and *Egr3* in the progression of leukemia

**Fig. 1:**
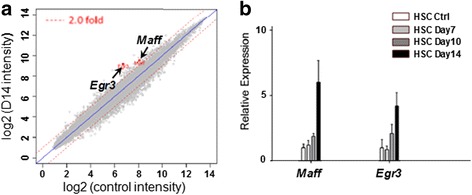
Differential expression profiles of *Maff* and *Egr3* during leukemia progression. **a** According to microarray data, *Maff* and *Egr3* are significantly highly expressed under leukemia. Their expressions reach the highest levels at the late stage (≥ Day 14) of leukemia, which are shown herein. The x- and y- axes are the log_2_ values of gene expression intensities in the microarray (leukemia and control, respectively); and the *two red* dashed lines (parallel to the diagonal) represent expression levels that are of 2-fold changes comparing to control, which serve as conventional indicators for significant changes in gene expression. **b** Relative expression levels of the two genes measured by qRT-PCR are shown. The data validate the high expressions of the two genes in HSCs under leukemia

As done in our previous research, we profiled the gene expressions in BM HSCs (from control and leukemia mice) at different time points (Day 7, Day 10 and Day 14) and screened the differentially expressed genes [7, 8]. Since highly-expressed genes were usually regarded as having biological importance [14], we highlighted *Maff* and *Egr3* as they were among the most highly expressed (Fig. 1a) and their functional roles in HSCs under leukemia were less known [7, 8]. The high expressions of the two genes in HSCs were validated using quantitative reverse-transcription PCR (qRT-PCR) (Fig. 1b).

### System modeling revealed the molecular interaction mechanisms of *Maff* and *Egr3* in HSC cell cycle

**Fig. 2:**
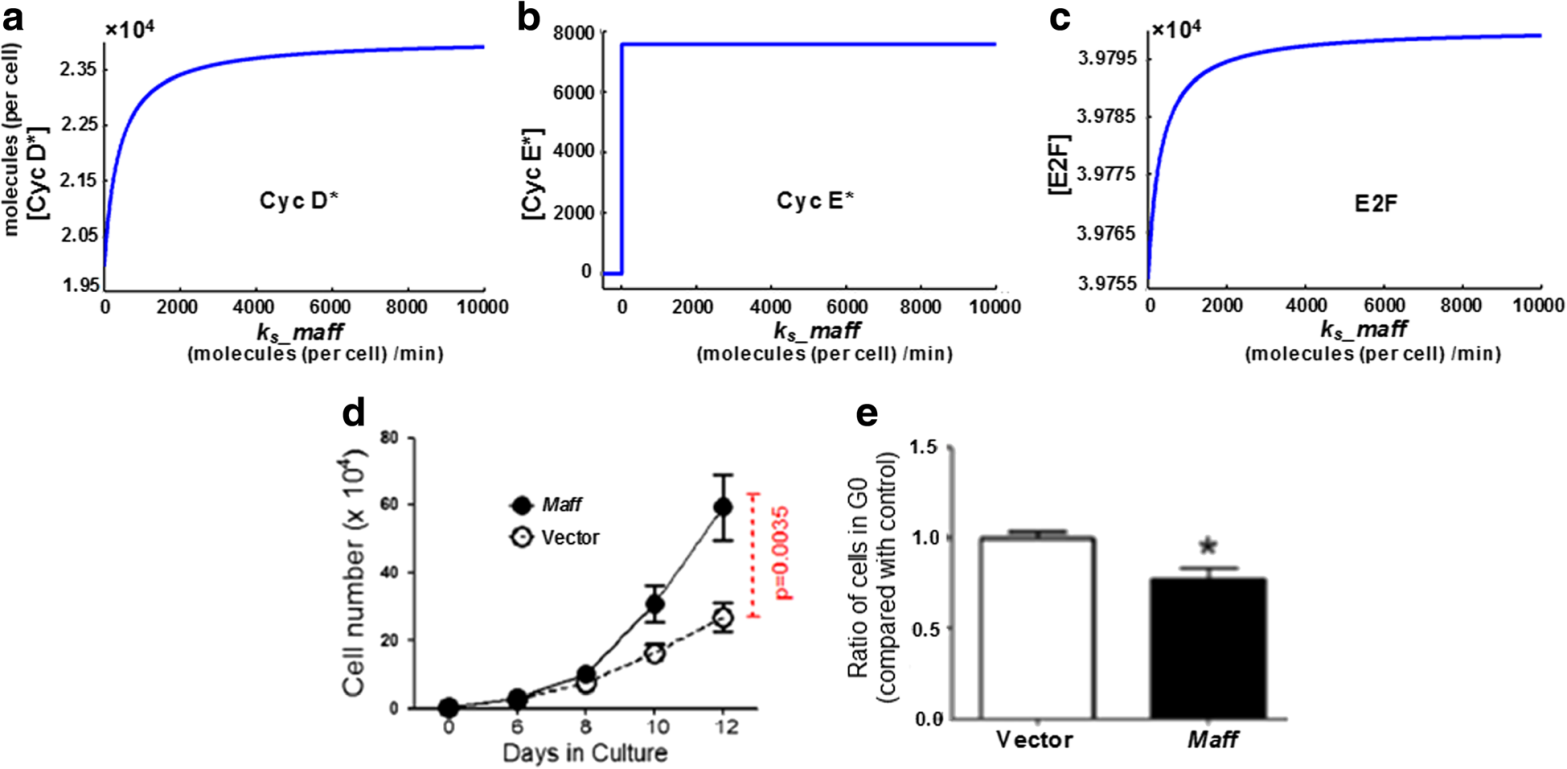
Simulations of system dynamics with respect to *Maff* expression. **a**-**c** Stable equilibriums (steady states) of Cyc D*, Cyc E* and E2F with respect to the expression level of *Maff* are shown in (**a**), (**b**) and (**c**) respectively. As known, the three molecules are benchmarks for the transitions of G0 - > G1/G1 - > S in cell cycle and their levels directly correspond to the speeds that cells proceed through the transition checkpoints. The data curves consist of the steady state concentrations of the molecules under various genetic synthesis rates (*k*_*s*__*maff*, i.e. expression level) of *Maff*. As shown herein, steady states of Cyc D* **a** and E2F **c** have increased as *k*_*s*__*maff* rises, indicating that G0 - > G1 is accelerated upon high expression of *Maff*. In addition, Cyc E* **b** is maintained at a level that is capable of driving G1 - > S. Cyc D/E* means the activated forms of Cyclin D/E (i.e. being bound with their corresponding Cdks). Metric units of x- and y- axes are “moles/cell/min” and “moles/cell”. **d** Proliferation of HSCs over-expressing (i.e. transducted with) *Maff* is shown herein; “Vector” means cell transducted with an empty vector (i.e. control data). As shown, the proliferation capability of HSCs with high expression of *Maff* is significantly larger (*p*-value <0.005 compared with control). **e** Ratio of HSCs staying in G0 phase before (control) and after *Maff* transduction (over-expression). Cells in different cycling status are sorted using flow cytometry according to Ki67 and Hoechst 33,342 staining. Data show that the ratio of G0 HSCs is significently lowered when *Maff* is highly expressed (*p*-value <0.05), indicating that high *Maff* expression mobilizes HSCs to escape the G0 phase, thus more HSCs enter the later cycling phase(s)

**Fig. 3:**
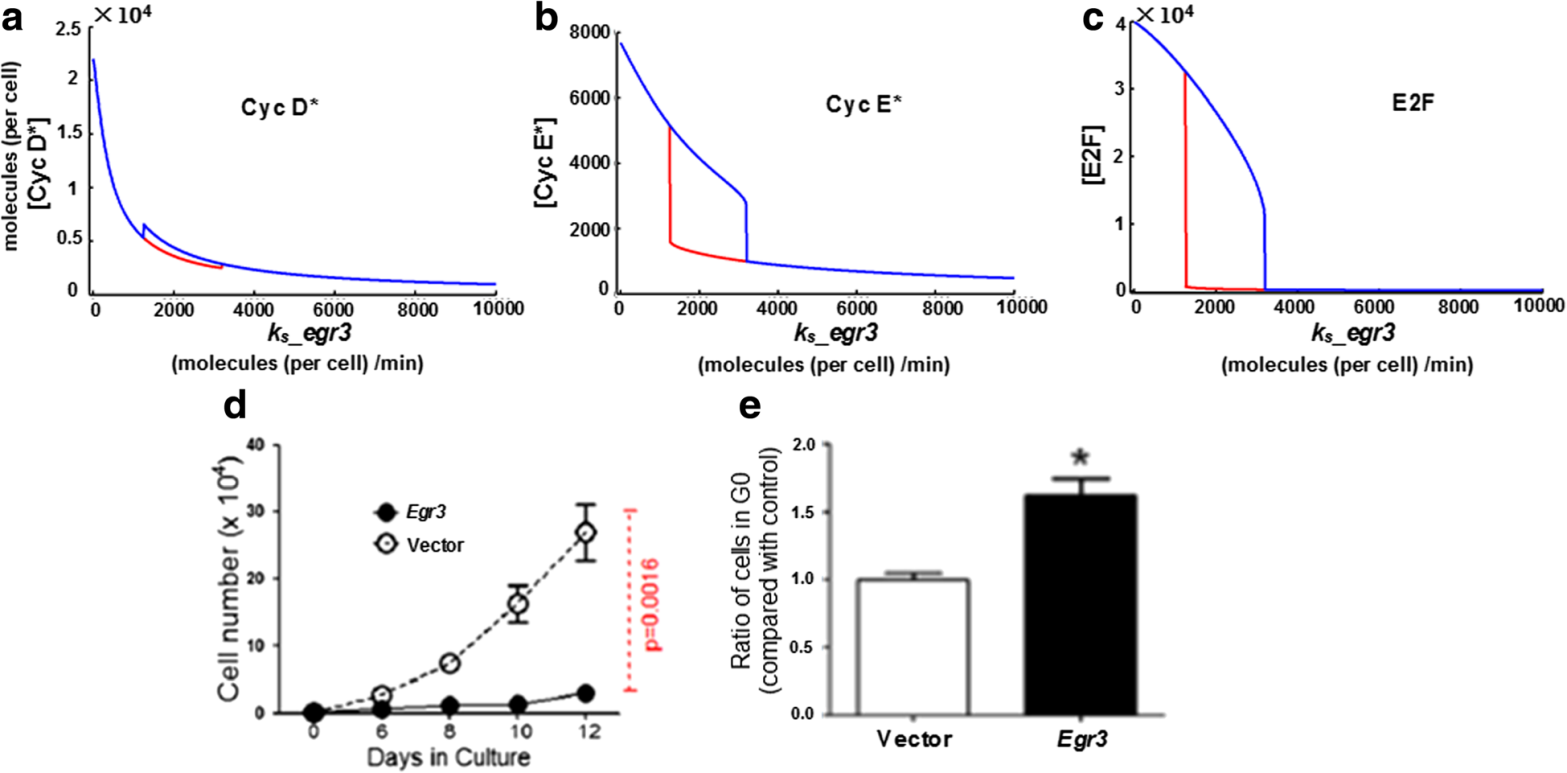
Simulations of system dynamics with respect to *Egr3* expression. **a**-**c** Stable equilibriums (steady states) of Cyc D*, Cyc E* and E2F with respect to the expression level of *Egr3* are shown in (**a**), (**b**) and (**c**) respectively. Similar to Fig. 2a–c, data are steady state concentrations under various genetic synthesis rates (*k*_*s*__*egr3*, i.e. expression level) of *Egr3*. Stable equilibriums of all the three cell cycle checkpoint-determining molecules have shifted downward as *k*_*s*__*egr3* increases, especially for Cyc E* **b** and E2F **c** as their steady states decline sharply in the manner of bistability (i.e. low-level stable equilibriums occur due to dynamical bifurcation; *red lines*). The steady states of Cyc D* exhibit very slight upward fluctuations at certain *Egr3* expression levels because the decreases in E2F at those *k*_*s*__*egr3* values result in less amounts of p18 and p19 (inhibitors of Cyc D*), thus causing the transient elevation. However, the Cyc D* level rapidly declines as *k*_*s*__*egr3* further increases. Noteworthy, when *k*_*s*__*egr3* exceeds certain values, the three molecules are all suppressed to very low levels, indicating that high *Egr3* expression potently suppresses cell cycle. **d** Proliferation of HSCs over-expressing (i.e. transducted with) *Egr3*; similar to Fig. 2d, “Vector” stands for control data. As shown, the proliferation capability of HSCs highly expressing *Egr3* is significantly lowered (*p*-value <0.005). **e** Ratio of HSCs staying in G0 phase before (control) and after *Egr3* over-expression. Data show that the ratio of G0 HSCs is significently heightened when *Egr3* is highly expressed (*p*-value <0.05), indicating that high *Egr3* expression arrests HSCs in the G0 phase, reducing the number of HSCs entering the later phase(s) of cell cycle

**Fig. 4:**
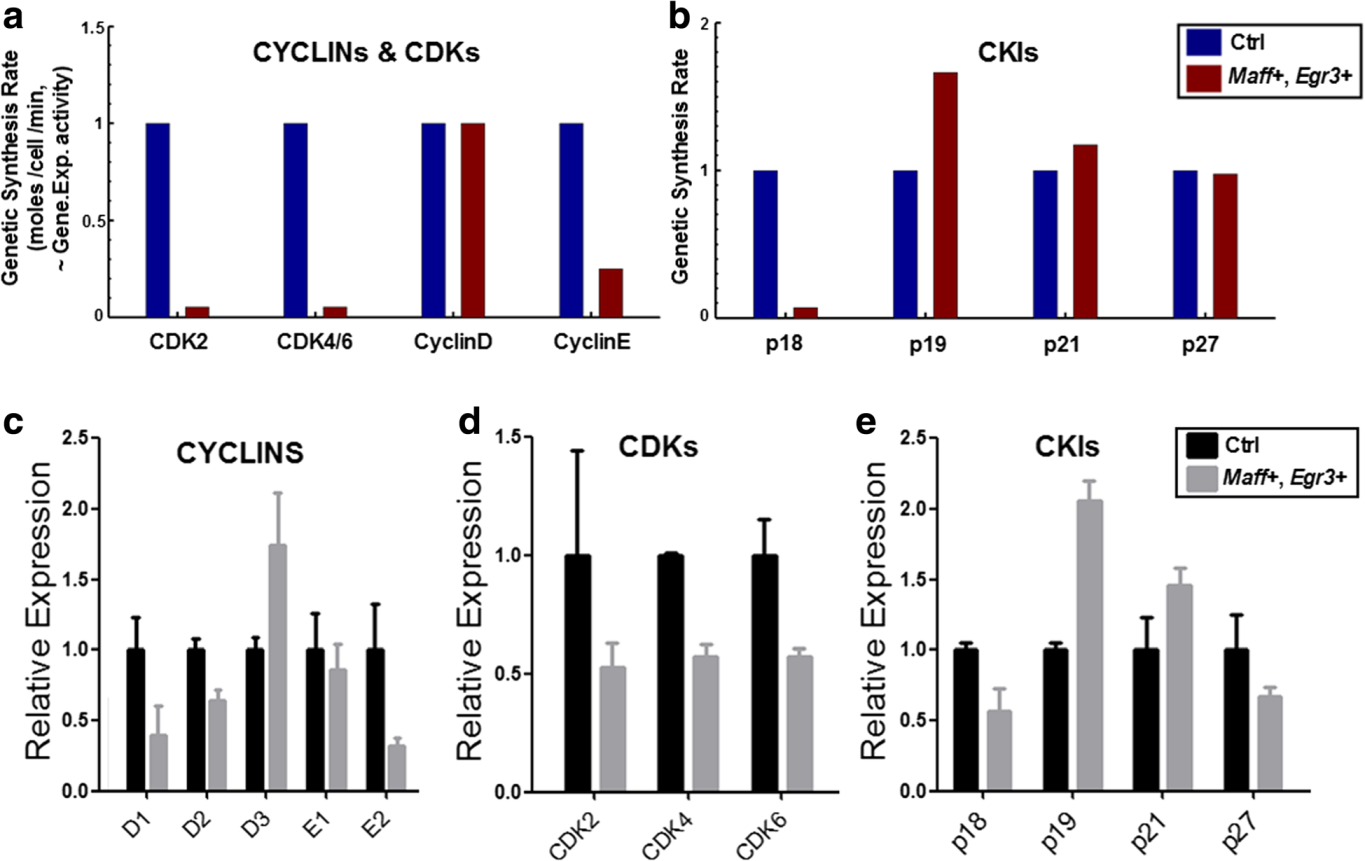
Simulations of gene expression activities of cell cycle components. **a**-**b** Genetic synthesis rates, which are proportional to the transcription rates of genes, are computed herein as surrogates for gene expression activities. Simulation data of Cyclin D, Cyclin E, Cdk2 and Cdk4/6 (**a)** and multiple CKIs (**b)** under the circumstance that *Maff* and *Egr3* are both highly-expressed (*Maff* ^+^, *Egr3* ^+^) are shown. Subtypes of cyclins are not distinguished in modeling in order for simplifying computation (i.e. Cyclin D1, D2 and D3 are taken altogether as one molecule Cyclin D; E1 and E2 are taken together as E). In the labels, “Gene.Exp.” means gene expression, “~” means equivalency or proportionality. **c**-**e**: Experimental measurements of relative expressions of the cyclins (**c**), Cdks (**d**) and CKIs (**e**) when *Maff* and *Egr3* are both highly-expressed (*Maff* ^+^, *Egr3* ^+^; i.e. Day 14 of leukemia). Data are obtained by qRT-PCR; and it is shown that the simulation results are consistent with experimental data in terms of expressions of all CKIs, Cdks and Cyclins. Since the modeling does not distinguish molecular subtypes, simulation of Cyclin D expression amounts to the overall level of all subtypes, which is also consistent with the sum of D1, D2 and D3 in the experimental data

**Fig. 5:**
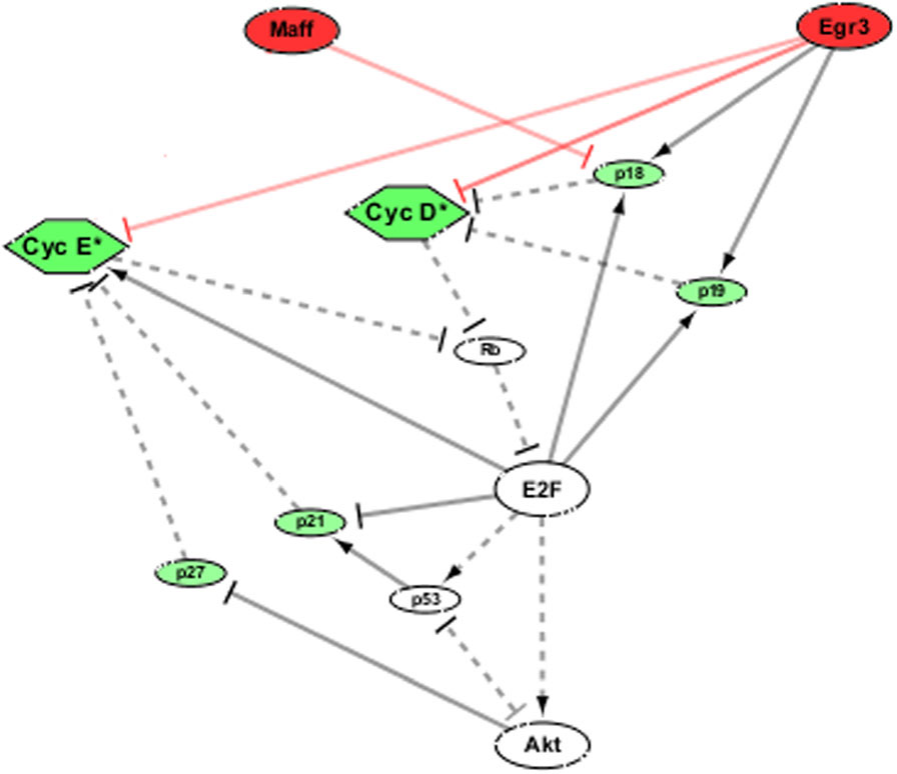
Molecular regulatory system of cell cycle (G0 → G1/G1 → S). Since the HSCs in which *Maff* and *Egr3* are highly expressed (i.e. functional) are mainly G0-phase cells (over 90%), it implicates that if *Maff* and *Egr3* have influence(s) on cell cycle, they must exert influence(s) in G0 → G1/G1 → S in the first place. Therefore, as a preliminary attempt, we summarized all key regulators in G0 → G1/G1 → S (including two cyclins, five CKIs, Rb, E2F and Akt) and examined all possible ways that *Maff* and *Egr3* (highlighted in red) act on the molecular network that result in consistent phenomena with experimental observations. Symbols: “−⊣” - inhibition; “―→” - activation; *solid lines* - transcriptional regulations; *dashed lines* - biochemical interactions; *red lines*: computionally inferred molecular regulatory relationships mediated by *Maff* and *Egr3*

Although bioinformatic databases such as NCBI, REACTOME, and KEGG suggested that *Maff* and *Egr3* collocate with several cell cycle components (e.g. Cdk2, Cdk4/6, etc.) in low-level, specific functional pathways involved in transcription regulations of hematopoietic differentiation/development, they did not include any knowledge about the molecular mechanisms that how *Maff* or *Egr3* influenced cell cycle [15, 16]. Since systems biology implied that components co-existing in a specific bio-pathway were functionally coordinated [17], it could be fairly assumed that there might be (direct or indirect) functional relations between the two genes and the cell cycle components. We systematically implemented in silico tests on combinations of all possibilities that *Maff*/*Egr3* had positive, negative or no actions at all, on the cell cycle components (Additional file 7: Table S1), aiming to examine which ways of actions formed a system structure that gave rise to the particular kinetic phenomena observed in experiments. We used the experimental data of in vitro liquid culture, cell cycle flow cytometry and gene expressions of HSC cell cycle as references [7]. From the in silico tests, we proved that at least three molecular actions that “*Maff* − ⊣ *p18*”, “*Egr3* − ⊣ Cdk2(:CyclinE)”, and “*Egr3* − ⊣ Cdk4/6(:CyclinD)” were necessary for the experimental phenomena that (individual) transductions (i.e. overexpressions) of *Maff* and *Egr3* greatly accelerated and suppressed HSC cell cycle (Figs. 2 and 3), as well as the real expression levels of cell cycle regulators in HSCs which both *Maff* and *Egr3* were highly expressed (Fig. 4). The three molecular actions formed a minimum inference of the mechanisms that *Maff* and *Egr3* functionally influenced the HSC cell cycle (Fig. 5, Additional files 1, 2, 3: Figure S1, S2, S3, Additional file 7: Table S1). For other ways of actions, in clear contrast to Figs. 2, 3, 4, the system structures they dictated could not generate the observed kinetics; thus they were discarded as false hypotheses in model selection (Additional file 4: Figure S4). For details of methods, refer to Materials and Methods and Additional file 6.

### Model-based computational analyses of the dynamics of *Maff*/*Egr3*- mediated alterations of HSC cell cycle under leukemia

**Fig. 6:**
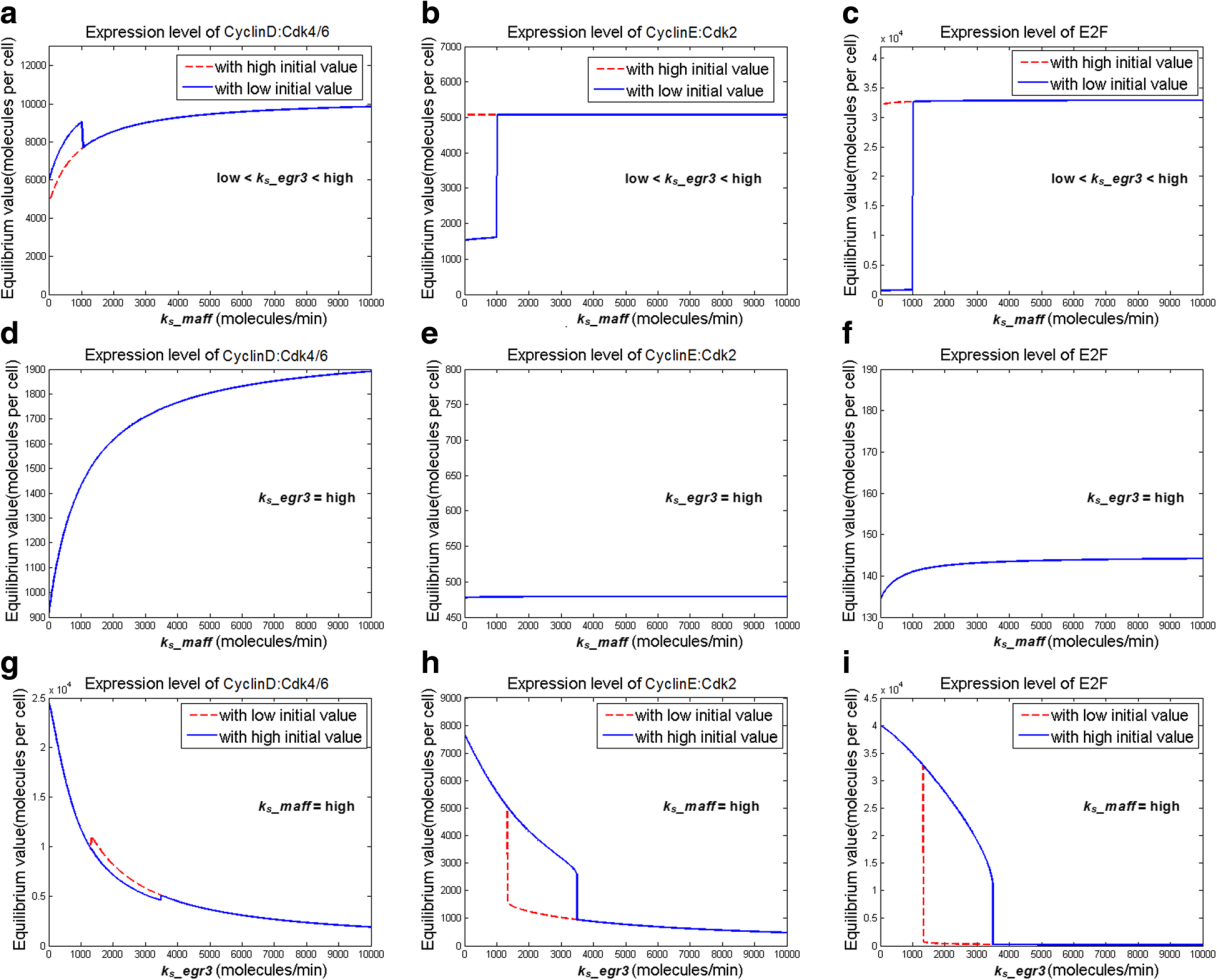
Dynamic simulations of *Maff* and *Egr3*-mediated alterations of cell cycle. **a**-**c** Steady states of Cyc D* (**a**), Cyc E* (**b**) and E2F (**c**) with respect to *k*_*s*_ *_maff* under medium *Egr3* expression level (low < *k*_*s*__*egr3* < high) are shown herein. In this circumstance, higher *Maff* expression is able to uplift the levels of Cyc D*, Cyc E* and E2F, resulting in bistability in their steady states (*red lines*). Dynamics of the three molecules under low *Egr3* expression level (with respect to *k*_*s*_ *_maff*) are equivalent to Fig. 2a-c, in which high expression of *Egr3* is not presumed (*k*_*s*__*egr3* = low). **d**-**f** Steady states of Cyc D* (**d**), Cyc E* (**e**) and E2F (**f**) with respect to *k*_*s*_ *_maff* under high *Egr3* expression level (*k*_*s*__*egr3* = high). In this circumstance, increase in *Maff* expression (*k*_*s*_ *_maff*) is unable to effectively uplift the steady states of Cyc D*, Cyc E* and E2F any more. **g**-**i** Steady states of Cyc D* (**g**), Cyc E* (**h**) and E2F (**i**) with respect to *k*_*s*_ *_egr3* when *Maff* expression level is high (*k*_*s*_ *_maff* = high). The situation is similar to those of Fig. 3a - c (*k*_*s*_ *_maff* = low), in which high *Egr3* expression potently suppresses the steady-state levels of all three molecules. The results indicate that no matter *Maff* expression is high or low, cell cycle is suppressed when *Egr3* is highly expressed

Based on the model structure endowed by the three molecular actions “*Maff* − ⊣ *p18*”, “*Egr3* − ⊣ Cdk2(:CyclinE)”, and “*Egr3* − ⊣ Cdk4/6(:CyclinD)”, we can further explore the regulatory mechanisms of *Maff* and *Egr3* computationally. By dynamical analyses, we observed that the expression of *Maff* generated a bistable system state to accelerate cell cycle only when *Egr3* expression was low or medium (Figs. 6a-c). On the other hand, when *Egr3* was highly expressed, no matter *Maff* expression was high or low, cell cycle was not accelerated as the system could not yield a bistability that increased the levels of the cell cycle checkpoint-determinants (Figs. 6a-i). The results indicated that although *Maff* and *Egr3* had opposite regulatory effects on cell cycle, the inhibitory power of *Egr3* was more potent than the activatory ability of *Maff*.

### Validation of the molecular actions by analyses of gene sequences

To further confirm the fidelity of the study and minify the possibility of overfitting in modeling, for each discovered molecular action (molecule *X* genetically acting on *Y*), we validated it by examining if the gene sequence of *Y* harbored any regulatory element on which the gene product of *X* could act, or if there existed an (or more) intermediate(s) *Z*, who could act on *Y* and harbored target regulatory elements of *X* at the same time.

**Fig. 7:**
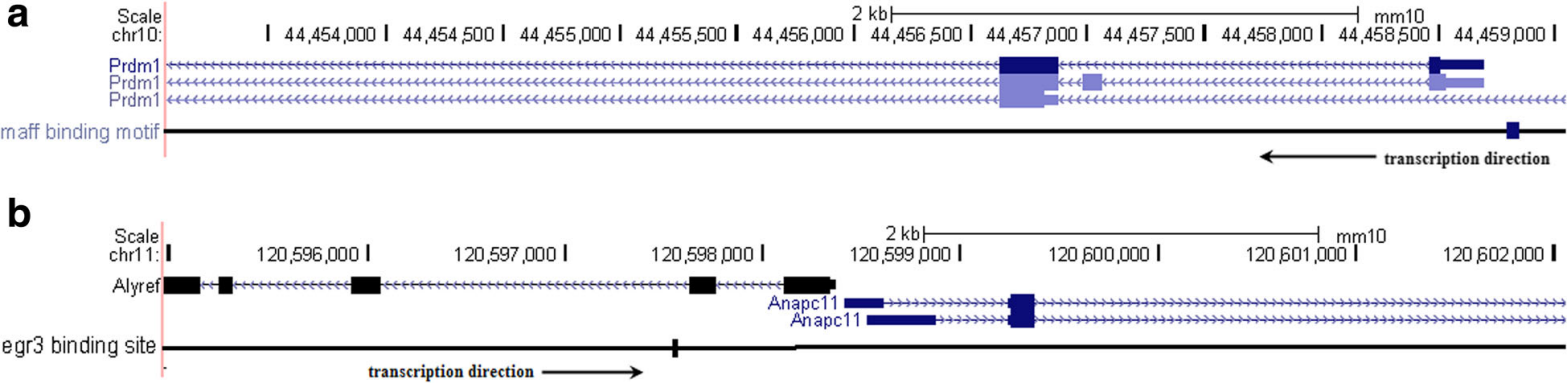
Validation of the molecular interactions by sequence analysis. **a** According to our survey of gene sequences, *p18* does not harbor the direct recognition element (i.e. binding motif) of Maff in its transcription promoter region; therefore, we check if there is any intermediate that both contains that motif (in the promoter region) and can negatively regulate *p18*. We have found that *Blimp1*/*Prdm1*, which is also a transcription factor in the regulation of hematopoietic differentiation, harbors the Maff binding motif in its promoter region; and the motif is 156 bp upstream the transcription start site (TSS). Meanwhile, *Blimp1*/*Prdm1* can transcriptionally repress *p18* in regulation of the cycling of hematopoietic cells. Thus the transcription regulatory relation “*Maff* ― → *Blimp1* − ⊣ *p18*” is indicated, and our computational inference “*Maff* − ⊣ *p18*” is well supported. **b** Our survey has also identified that *Anapc11*, which is a core coding gene of the anaphase-promoting complex/cyclosome (APC/C) that inhibits mitotic cyclins and greatly suppresses G0 → G1/G1 → S, links to the cis element that Egr3 recognizes and exerts transcription activation (523 bp upstream the TSS). Therefore, it implies that “*Egr3* ― → *Anapc11*” and “Anapc11 −⊣ Cyclin D/Cdk4/6, Cyclin E/Cdk2”; thus our computational inference that “*Egr3* − ⊣ CyclinD:Cdk4/6, CyclinE:Cdk2” is supported

As it was known that Maff possessed a conserved basic region flanked by a heptad repeat motif (bZip), through which the protein recognized a specific palindromic sequence TGCTGAC(G)TCAGCA (maf recognition element, MARE) to mediate DNA binding and potentiate gene transcription [18]. Since the *p18* gene did not contain MARE, we surveyed all known genes that contained MARE in the promoter region (i.e. in a range of ~2kbp upstream TSS, by convention). We identified that *Blimp1/Prdm1* contained MARE at the location of 156 bp upstream its TSS (Fig. 7a); meanwhile, *Blimp1* was shown to repress *p18* expression by various studies [19, 20]. Furthermore, *Blimp1* was also a transcription factor of hematopoiesis functioning in differentiation of blood cells, thus coherently relevant to cell cycle regulation. In all, Maff could act as transcription factor on (i.e. activate) *Blimp1*, and *Blimp1* subsequently down-regulated *p18*. Therefore, the acting relationship “*Maff* − ⊣ *p18*” had been confirmed.

For *Egr3*, we carried out the similar gene sequence survey to detect if it was functionally related with the cell cycle regulators (Cdk2/4/6, Cyclin D/E). We identified that gene *Anapc11* linked to a cis element (CGCCCCCGC) which Egr3 could recognize and activate transcription (Fig. 7b). The product of *Anapc11*, APC11, was a core catalytic subunit of the anaphase-promoting complex/cyclosome (APC/C), which could act on mitotic cyclins and greatly suppress the transition of G0/G1 phase to S phase [21]. Since it was CyclinD:Cdk4/6 and CyclinE:Cdk2 that governed the phase transition G0/G1 → S and Egr3 could transcriptionally activate APC11, it was a support for our inferences that the acting relationships “*Egr3* − ⊣ CyclinD:Cdk4/6” and “*Egr3* − ⊣ CyclinE:Cdk2” might exist. In all validations, all molecules surveyed originated from the same species as our study subject (mouse).

### Mechanistic implications of high *Maff* and *Egr3* expressions in hematopoietic molecular regulations

One would intuitively assume that since *Maff* stimulated cell cycle (and thus proliferation) of HSCs, HSCs might highly express it so as to adapt to the leukemic environment, where the surrounding malignant cells were highly proliferative. It was no surprise that normal cells were apt to be cancerized in a malignant environment, since adaptation was an universal cellular behavior from bacteria to higher organisms [22, 23].

**Fig. 8:**
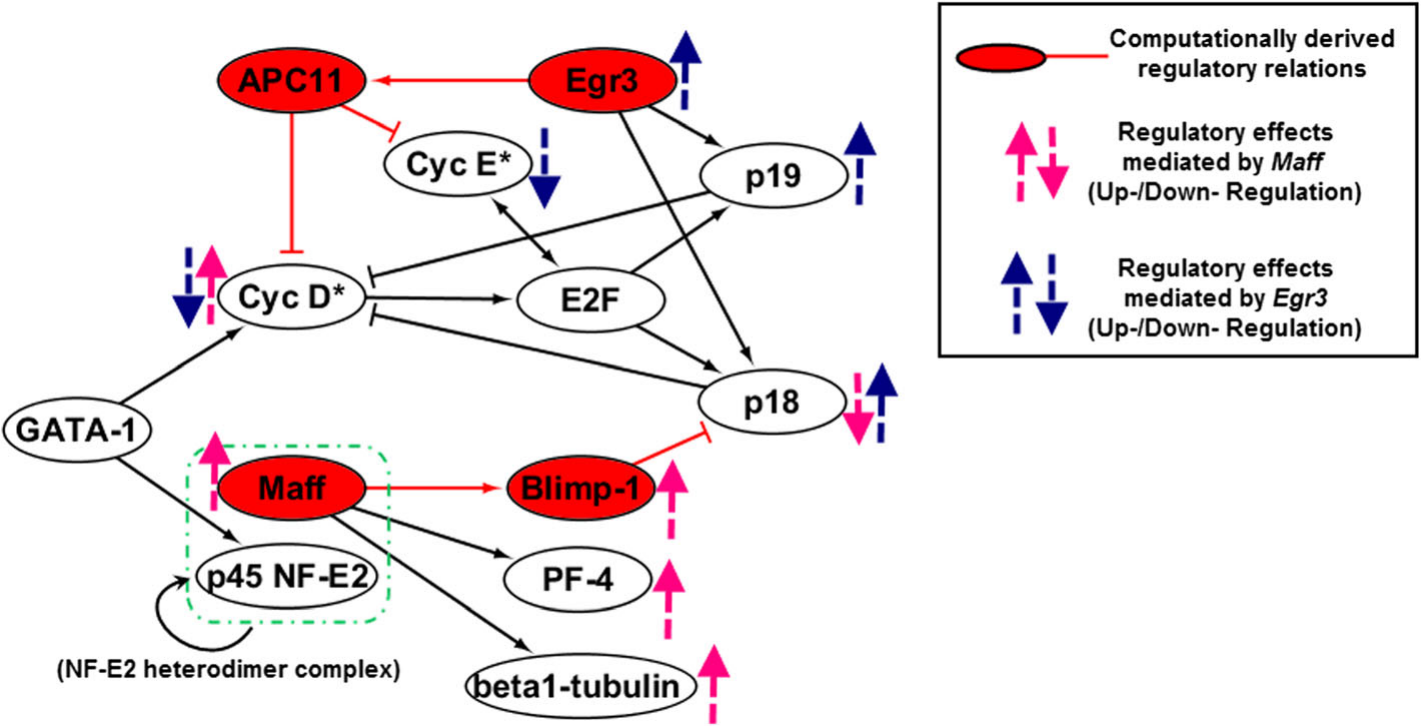
Molecular regulations of cell cycle and hematopoiesis. GATA-1 (*Gata-1*) activates the cell cycle components as well as p45 NF-E2, the heterodimer partner of Maff. The Maff:p45 NF-E2 functional complex is self-regulated and it activates the transcriptions of various downstream regulators, e.g. Blimp-1 (*Blimp1*), β1-tubulin (*Tubb1*), and PF-4 (*Pf4*). Altogether with our prediction that “*Maff* − ⊣ *p18*”, it can be concluded that high expression of *Maff* eliminates the feedback inhibtion of p18 to Cyclin D:Cdk4/6, as well as up-regulating the downstream TFs. Hence, cell cycle will be accelerated and proliferation/differentiation of hematopoietic cells are enhanced. This is why we hypothesize that high expression of *Maff* adapts cells to a rapidly proliferative status, i.e. cancerization. On the other side, *Egr3* suppresses the checkpoint controllers of G0 → G1/G1 → S and activates CKIs, thus its high expression greatly inhibits cell cycle. Thus it is fairly assumed that it may be a “harsh control” via which HSCs forcibly shut down functionalities when their behaviors exceed the limits of cellularity controlling principles, i.e. self-protection

However, the molecular mechanisms of why the actions of *Maff* (“*Maff* ⟶ *Blimp1* − ⊣ *p18*”) were necessary for the cancerization of HSCs, remained unclear. We answered the question by depicting the hematopoietic molecular network of important transcription regulators with our newly revealed knowledge (Fig. 8). In mammalian, GATA-1 (encoded by gene *Gata1*) was recognized as a master regulator at the upstream of hematopoietic differentiation; it transcriptionally activated various downstream regulators, including Cyclin D(1) and NF-E2/p45, the heterodimic functional partner of Maff [24, 25]. When Maff was highly expressed and gained the regulatory power upon activation of NF-E2/p45, it possibly activated transcriptions of other downstream regulators like Blimp-1 (*Blimp1*), β1-tubulin (*Tubb1*). In addition, we also identified that PF-4 (*Pf4*), another downstream regulator, harbored at its gene promoter region the transcription factor recognition motif for Maff (Additional file 5: Figure S5). In fact, these were all differentiation-promoting transcription regulators that were ought to be activated at later stages of HSC differentiation in normal condition [3, 26]. On the other hand, the suppression of *p18* by (high expression of) *Maff* breaks the negative feedback control on Cyclin D(:Cdk4/6), which amounts to a great up-regulation of Cyclin D activity. Since Cyclin D is the initiating regulator of cell cycle, thus high expression of *Maff* accelerates the overall cell cycle. Therefore, for sake of sufficiently accelerating cell proliferation, high expression of *Maff* became necessary for both transcriptionally repressing the CKI and potentiating the downstream regulators, as a means of adapting to the malignant environment.

The functional role of *Egr3* was more clear. It had already been experimentally revealed that *Egr3* up-regulated *p18* and *p19* expressions [7]. However, although the two CKIs biochemically inhibited the activity of Cyclin D(:Cdk4/6), we computationally discovered the inhibitions alone were not sufficient for potent suppression of cell cycle (Additional file 4: Figure S4A-C). To realize the largely-increased G0 ratio of HSCs as experimentally observed (Fig. 3e), it was necessary that *Egr3* genetically suppressed CyclinD:Cdk4/6 and CyclinE:Cdk2. Therefore, it could be fairly supposed that *Egr3* was highly expressed to suppress cell cycle to fullfil the cellularity controlling principles when proliferations or demands for resources exceeded certain limits. Based on the above, we preliminarily explained why the two functionally opposite genes *Maff* and *Egr3* were both highly expressed in the leukemic environment (especially at the late stage, i.e. ≥ day 14).

## Discussion

In our previous study, we identified that *Maff* and *Egr3* were two highly-expressed genes which were important to hematopoiesis and had opposite functional influences on (BM) HSC cell cycle. However, the molecular mechanisms of how they mediate the cell cycle alterations remain unrevealed. By utilizing bioinformatics resources and systems biology approaches, we summarized all database-registered molecular relationships and identified the (minimal set of) molecular actions that lead to the very experimentally-observed cell cycle kinetics and gene expressions, via testing all possible hypothese of molecular actions (i.e. model selection) in a curated mammalian cell cycle kinetic model [11–13]. We have in silico demonstrated that the three regulatory relationships we identified are at least required, the experimental observation cannot be reproduced without any one of them (Additional file 4: Figure S4).

It is interesting to ponder why the two functionally opposite genes are both highly expressed in leukemia. Since *Maff* stimulates cell cycle by down-regulating *p18*, its high expression is possibly resulted from adaptation to the malignant environment (i.e. cancerization), in which leukemic cells rapidly proliferate. However, we sample the normal HSCs from a non-irradiated animal model, thus their normal physiological/biophysical properties (i.e. controlling mechanisms of cellularity) are kept [7]. Therefore, although they have the ability of adaptation, these HSCs cannot become completely cancerous after all. Hence, it is fairly assumed that when proliferations or demands for resources exceed certain limits, cells forcibly shut down functionalities to fullfil the cellularity controlling principles. Since overexpression of *Egr3* potently suppresses cell cycle (Fig. 6), it is possibly a “harsh control” measure that HSCs employ in leukemic environment.

Furthermore, the relativity of *Maff*/*Egr3* functions for HSC cell cycle can only be screened with a non-irradiated (i.e. un-manipulated) leukemia model, as intact HSCs without overt damages (e.g. immune system destruction) can be measured during leukemia progression. On the contrary, given the typical pre-conditioned leukemia models utilized in most of previous studies, the physiology of native leukemogenesis is destroyed, i.e. non-leukemic cells in the leukemic host are no longer the “normal” cells because they are heavily damaged by the myeloablative manipulations and thus deviant far from the normal cellularity [7, 9, 27–30]. Gene expressions in those study designs (other than ours) cannot represent in vivo functions; hence if in those cases, *Maff*/*Egr3* regulations on HSC cell cycle could not have been inferred.

In our previous research, we found that in leukemic BM, the HPC count increased and the rate that HSCs differentiate into HPCs was high at the beginning; meanwhile, the differentiation of HSCs towards HPCs was almost shut off later and the HPC count decreased acutely after day 14 [9]. Furthermore, significantly increased quiescence of HSCs was observed at the late stage of leukemia [7]. These cellular-level results render a hint that HSCs respond to leukemia with a process of cancerization/self-protection. When leukemia emerges, HSCs accelerate their cell cycles to proliferate into more functional blood cells to compensate cell loss; on the other hand, as leukemic cells become dominant and outgrow normal cells, most HSCs stay in the quiescent state, which is far less sensitive to environmental affects [7, 9, 31, 32]. Here in this study, by revealing how *Maff* and *Egr3* act on cell cycle, we find the trait of “cancerization/self-protection” on the molecular level. Thus this pilot finding may enhance the suggested mechanism and provide a further explanation for the response of HSCs to leukemic environment.

In methodology, we assembled bioinformatic resources of molecular interactions and refined them by kinetic modeling to acquire pilot knowledge. As bioinformatics generates likelihoods and kinetic modeling provides explanations for mechanisms, our approach presented herein sets a preliminary model for the combined efforts. Such a practice is worthwhile to be generalized and extended for further utilities.

## Conclusion

Previous studies have shown that functions of normal HSCs are altered in leukemic environment. Unexpectedly, two genes *Maff* and *Egr3* that are originally regarded as functioning mainly in somewhere else rather than HSCs, are important for the functional alteration. Moreover, the two genes oppositely function on cell cycle but they are both highly expressed under leukemia. Due to the interest to study the functional alteration of HSCs as well as the difficulty for in vivo experiments, we combine computational and experimental approaches to investigate the genes’ acting mechanisms on cell cycle. We have identified three potential molecular regulations and the results indicate that HSCs tend to adapt to the malignant environment but eventually shut down the functionality to stay in the dormant state. These results may provide insights for future studies; and moreover, our combined use of experimental/computational efforts is also applicable to other studies and inspires interdisciplinary research methods.

## Methods

### Experimental animal model of AML

The non-irradiated mice models of leukemia and control were established according to the protocols described in the previous work [7]. All mice were maintained in the animal facility and samples of BM HSCs (CD45.1^+^LKS^+^) were extracted from both leukemia and control mice at different time points (days: 0, 7, 10 and 14) for further measurements.

### Microarray analysis

Microarray of gene expressions in the CD45.1^+^LKS^+^ cells was performed at CapitalBio in Beijing. Procedures for total RNA extraction, cRNA amplification, labeling, and hybridization, as well as RNA quality confirmation, were implemented according to protocols that we described previously [7, 8]. Raw data normalization and analysis of differentially-expressed genes were done using Microarray Analysis Software v5.0 (Affymetrix).

### Quantitative reverse-transcription PCR (qRT-PCR)

Total RNA was isolated using Qiagen RNeasy mini kit. cDNA was synthesized using Improm-II™ reverse transcriptase (Promega) or SuperScrip™ III (Invitrogen). qRT-PCR was carried out with primers/probes that were previously specified [7, 8]. qRT-PCR was performed on 7500 or StepOne real-time PCR system (Applied Biosystems).

### Flow cytometry

HSCs were flow sorted by gating on Lin^−^ Sca-1^+^ c-Kit^+^ (LKS^+^) of the CD45.1^+^ cells from the BM sample of our mouse model. Cell cycle states of HSCs (LKS^+^) were recognized by the marker of cytoplastic protein Ki67 and addition of DNA dye Hoechst33342. Cells in G0 phase were gated on Ki67^−^ Hoechst^−^; cells in G1 phase were gated on Ki67^+^ Hoechst^−^; and cells in S/G2/M phase were gated on Ki67^+^ Hoechst^+^. All antibodies (Abs) used herein were from BD Biosciences or e-Bioscience; and all experimental procedures were implemented according to the protocols that we specified previously [7].

### Transduction of HSCs

The *Maff* and *Egr3* cDNAs were purchased from Origene and HSCs (CD45.1^+^LKS^+^) were transfected with retroviruses containing plasmids constructed with the *Maff* and *Egr3* cDNAs or an empty segment (control). Plasmid construction and retroviruse production were according to protocols previously described [7, 8]. Cells were pre-cultured for 1 day, then transfected with retroviruses (*Maff*, *Egr3* or control) and incubated for another 2 days. The culture was maintained in StemSpan SFEM medium. After transduction, GFP^+^ cells were sorted for assays.

### In vitro liquid culture

1000 transduced cells (with *Maff*, *Egr3* or control; GFP^+^) were sorted into 16 wells of a 96-well plate per group, cultured in SFEM medium. Cell numbers were counted every 2 days after day 6 using flow cytometry.

### Bioinformatic resources of molecular interactions

Data of molecular interactions and functional pathways were referenced from public databases of NCBI, REACTOME, and KEGG. Genes/proteins associating with *Egr3* and *Maff* as well as collocating with them in specific functional pathways of hematopoietic cell cycle regulations were surveyed as potential targets that *Maff* and *Egr3* acted on cell cycle. So far as G0 → G1/G1 → S were considered, there were three molecules, namely, Cdk2/CyclinE, Cdk4/6/CyclinD, and p18. Respectively, *Maff* was potentially associated with Cdk2/CyclinE and p18; and *Egr3* was suggested to be associated with Cdk2/CyclinE and Cdk4/6/CyclinD.

### Kinetic modeling of cell cycle (G0 → G1/G1 → S)

Ordinary differential equations (ODEs) were used to model all the genes’ dynamics in terms of expression levels. First-order Hill function was utilized to describe both regulations of gene transcription and protein activity. We considered the expression levels of Cdk2(:CyclinE), Cdk4/6(:CyclinD) and E2F to be the controlling benchmarks, because their expression levels were nearly invariant in cell cycle [13]. We also considered the coupling of cell cycle with cell apoptosis and survival (mediated by p53 and AKT, respectively). Parameters from various references were collected [12, 13, 33–38], in which transcription rates were scaled to the benchmarks. For all ODEs, their formulas were shown in Additional file 6 and values of parameters were listed in Additional file 8: Table S2.

### Model selection

We assumed that *Maff* and *Egr3* might have positive, negative or no actions at all, on their respective associating cell-cycle-related genes or proteins. We systematically implemented in silico tests on all combinations of the possibilities, which were 3^4^ = 81 combinations in total (Additional file 7: Table S1). For each combination (*Ω*_*i*_, *i* = 1,2,…,81) specified by possible ways that *Maff* and *Egr3* acted on Cdk2/CyclinE, Cdk4/6/CyclinD and p18, i.e.

$$ {\displaystyle \begin{array}{l}{\varOmega}_i=\left\{ Maff\overset{i_1}{\to } Cdk2, Maff\overset{i_2}{\to }p18, Egr3\overset{i_3}{\to } Cdk2, Egr3\overset{i_4}{\to } Cdk4/6\right\}\\ {}\left({i}_k\in \left\{ positive, negative, no\right\},k=1,2,3,4\right)\end{array}} $$

the *in silico* test was simulating the dynamic states of the integrated model (*M*_*i*_) comprised by the cell cycle model and *Ω*_*i*_ (see Additional file 6 for details). We checked if the simulation results were consistent with the experimental observations of cell proliferation (in vitro liquid culture), G0-phase proportion (cell cycle flow cytometry) and cell-cycle-related gene expressions. Based on the tests, we could see which ways of actions formed a system structure that gave rise to the particular phenomena observed in experiments. These molecular actions were regarded as rational predictions and used for further analyses; the rest were discarded as false hypotheses.

### Dynamic simulation

With an initial value in the normal range of molecular quantity level [13, 33, 36–38], we obtained the time-courses of molecular quantities by numerically solving the ODEs (Additional file 6 and Additional file 8: Table S2). We used the Gear method so as to alleviate the stiffness problem of ODEs [39]. For comparison with experimental observations, stable equilibriums of (dynamic) system states must be computed, since it was the equilibriums, not the transient states in the midst of time-courses, were correspondent to experimental observations that could be stably measured (i.e. states that the system could steadily reside). If a system had equilibrium(s), its time-course trajectories tend to some area(s) over adequately large ranges of time and parameter spaces. And if it did not, trajectories spread out (usually traversing several orders of magnitudes). To locate the equilibrium, we utilized the state at the end time point of the time-course as an initial guess and used the trust-region method [40]. The stability of the equilibrium was defined following the concept of Lyapunov theory [41]. For an equilibrium, eigenvalues of the Jacobian matrix (shown below) evaluated at it were examined. If all eigenvalues had negative real parts, the equilibrium is (asymptotically) stable; if any of them had a positive real part, the equilibrium is unstable.

$$ {J}_{X_{eq}}={\left[\frac{\partial \left(A\cdot R\left(X,P\right)\right)}{\partial X}\right]}_{X={X}_{eq}} $$

### Gene sequence analysis

Sequence alignment was employed to detect whether a given gene harbored a TF-binding element. We adopted the most stringent criterion to require that all nucleotides must be matched so that the identification of an element was accounted. Such a practice might help avoid ambiguity in the standard of identification and filter out positive false results. Moreover, we only consider nucleotide segments in the range of 2kbp upstream from the TSS to be the positive identification of a regulatory element for a gene. All gene sequences surveyed herein were referenced from the NCBI Gene database.

## Additional files

Additional file 1: Figure S1. Regulatory network with edge “Maff → Cdk2 (:Cyclin E)”. Another possible regulatory model includes an additional positive regulation on Cdk2/CyclinE by Maff, which is also capable of reproducing the qualitatively correct dynamic profiles illustrated by the experimental data (see Additional file 7: Table S1 for details). Here we do not discriminate the correctness between the model shown in Fig. 5 and the alternative one herein, as both models are qualitatively valid given the current data. The minimal model shown in Fig. 5 is chosen as example due to the principle of simplicity. (PNG 31 kb)

Additional file 2: Figure S2. Simulation results with additional regulation “Maff → Cdk2 (:Cyclin E)” with respect to *Maff*. Dynamics with respect to the transcription rate of *Maff* at high (upper), medium (middle), and low (lower) *Egr3* expression-levels. In each panel, steady-state molecular quantities of Cyclin D-Cdk4/6 (left), Cyclin E-Cdk2 (middle) and E2F (right) are shown. The correct bistability with respect to *Maff* is qualitatively reproduced with the additional molecular action. (PNG 73 kb)

Additional file 3: Figure S3. Simualtion results with additional regulation “Maff → Cdk2 (:Cyclin E)” with respect to *Egr3*. Dynamics with respect to the transcription rate of *Egr3* at high (upper) and low (lower) *Maff* expression-levels. In each panel, steady-state molecular quantities of Cyclin D-Cdk4/6 (left), Cyclin E-Cdk2 (middle) and E2F (right) are shown. The correct bistability with respect to *Egr3* is qualitatively reproduced with the additional molecular action. (PNG 31 kb)

Additional file 4: Figure S4. Outcomes produced by other regulatory sturctures. Apparently false dynamics resulted by other hypotheses of regulations. Basically, all the other network structures than the one in Fig. 5/Additional file 1: Figure S1 produce qualitatively false results on (at least) one of Cdk4/6:CyclinD, Cdk2:CyclinE, and E2F. Here we show the most typically false results, combinations of regulatory relations are randomly assigned. Upper panel: unrealistic dynamic levels of Cdk2:CyclinE and E2F with respect to *Maff* transcription under low *Egr3* expression, which is dictated by a randomly assigned network structure (regulatory code 1212); middle panel: results of Cdk2:CyclinE and E2F dictated by another network structure (regulatory code 2133); results of Cdk4/6:CyclinD and E2F dictated by a third different network structure (regulatory code 3321). Refer to Additional file 7: Table S1 for depiction of the regulatory codes. (PNG 52 kb)

Additional file 5: Figure S5. Binding motif of Maff is also discovered within 2 kb upstream of *Pf4* gene. The Maff binding motif for transcriptional activation occurs at a location <1 kb upstream the transcription start site (TSS) of Pf4, which is potentially within the promoter region of the gene. The observation indicated that Maff might positively regulate Pf4, which is a regulator of platelet formation. (PDF 5 kb)

Additional file 6: Formulations of the mathematical model. Descriptions for the ODEs and the modeling process are enclosed here. (DOC 128 kb)

Additional file 7: Table S1. Table for the combinatorial numerical tests. Qualitative results of the numerical tests on combinations of possible regulatory relations are documented here, with all 81 combinations exhausted. The first four columns represent the regulatory code, “1” – inhibitory, “2” – none, and “3” – activatory effects, respectively. Molecular actions are indicated by the column headers. The last two columns show the agreement or discrepancy with input experimental data, “0” qualitative discrepancy, “1” qualitative agreement. The input experimental data for model training are the cell-cycle status after transduction of *Maff*/*Egr3* and qRT-PCR results for cell-cycle genes. (XLSX 13 kb)

Additional file 8: Table S2. Model parameters. Symbols, definitions, values, units and references for all model parameters are listed in the table here. (XLS 28 kb)

## Abbreviations

BM: Bone marrow
CDK: Cyclin-dependent kinase
CKI: cyclin-Dependent kinase inhibitor
HSC: Hematopoietic stem cell
MARE: Maf recognition element

## References

[1] Shivdasani RA, Rosenblatt MF, Zucker-Franklin D, Jackson CW, Hunt P, Saris CJM, Orkin SH (1995). Transcription factor NF-E2 is required for platelet formation independent of the actions of thrombopoeitin/MGDF in megakaryocyte development. Cell.

[2] Motohashi H, Katsuoka F, Shavit JA, Engel JD, Yamamoto M (2000). Positive or negative MARE-dependent transcriptional regulation is determined by the abundance of small Maf proteins. Cell.

[3] Shivdasani RA (2001). Molecular and transcriptional regulation of megakaryocyte differentiation. Stem Cells.

[4] Blok LJ, Grossmann ME, Perry JE, Tindall DJ (1995). Characterization of an early growth response gene, which encodes a zinc finger transcription factor, potentially involved in cell cycle regulation. Mol Endocrinol.

[5] Ogawa K, Sun J, Taketani S, Nakajima O, Nishitani C, Sassa S, Hayashi N, Yamamoto M, Shibahara S, Fujita H (2001). Heme mediates derepression of Maf recognition element through direct binding to transcription repressor Bach1. EMBO J.

[6] O'Donovan KJ, Tourtellotte WG, Millbrandt J, Baraban JM (1999). The EGR family of transcription-regulatory factors: progress at the interface of molecular and systems neuroscience. Trends Neurosci.

[7] Cheng H, Hao S, Liu Y, Pang Y, Ma S, Dong F, Xu J, Zheng G, Li S, Yuan W (2015). Leukemic marrow infiltration reveals a novel role for Egr3 as a potent inhibitor of normal hematopoietic stem cell proliferation. Blood.

[8] Cheng H, Liu Y, Jia Q, Ma S, Yuan W, Jia H, Cheng T (2016). Novel regulators in hematopoietic stem cells can be revealed by a functional approach under leukemic condition. Leukemia.

[9] Li R, Cheng H, Cheng T, Liu L (2016). Digitalization of a non-irradiated acute myeloid leukemia model. BMC Syst Biol.

[10] Sible JC, Tyson JJ (2007). Mathematical modeling as a tool for investigating cell cycle control networks. Methods.

[11] Csikász-Nagy A, Battogtokh D, Chen KC, Novák B, Tyson JJ (2006). Analysis of a generic model of eukaryotic cell-cycle regulation. Biophys J.

[12] Qu Z, Weiss JN, MacLellan WR (2003). Regulation of the mammalian cell cycle: a model of the G1-to-S transition. Am J Physiol Cell Physiol.

[13] Haberichter T, Mädge B, Christopher RA, Yoshioka N, Dhiman A, Miller R, Gendelman R, Aksenov SV, Khalil IG, Dowdy SF. A systems biology dynamical model of mammalian G1 cell cycle progression. Mol Syst Biol 2007;3(1). 10.1038/msb4100126 PMC1828753 17299420

[14] Okamura Y, Aoki Y, Obayashi T, Tadaka S, Ito S, Narise T, Kinoshita K. COXPRESdb in 2015: coexpression database for animal species by DNA-microarray and RNAseq-based expression data with multiple quality assessment systems. Nucleic Acids Res. 2014; 10.1093/nar/gku1163 PMC4383961 25392420

[15] Croft D, Mundo AF, Haw R, Milacic M, Weiser J, Wu G, Caudy M, Garapati P, Gillespie M, Kamdar MR (2014). The Reactome pathway knowledgebase. Nucleic Acids Res.

[16] Kanehisa M, Goto S (2000). KEGG: Kyoto Encyclopedia of Genes and Genomes. Nucleic Acids Res.

[17] Ideker T, Galitski T, Hood L (2001). A new approach to decoding life: Systems biology. Annu Rev Genom Human Genet.

[18] Yoshida T, Ohkumo T, Ishibashi S, Yasuda K (2005). The 5′-AT-rich half-site of Maf recognition element: a functional target for bZIP transcription factor Maf. Nucleic Acids Res.

[19] Vincent SD, Dunn NR, Sciammas R, Shapiro-Shalef M, Davis MM, Calame K, Bikoff EK, Robertson EJ (2005). The zinc finger transcriptional repressor Blimp1/Prdm1 is dispensable for early axis formation but is required for specification of primordial germ cells in the mouse. Development.

[20] Shaffer AL, Lin KI, Kuo TC, Yu X, Hurt EM, Rosenwald A, Giltnane JM, Yang L, Zhao H, Calame K (2002). Blimp-1 orchestrates plasma cell differentiation by extinguishing the mature B cell gene expression program. Immunity.

[21] Baeg GH, Matsumine A, Kuroda T, Bhattacharjee RN, Miyashiro I, Toyoshima K, Akiyama T (1995). The tumour suppressor gene product APC blocks cell cycle progression from G0/G1 to S phase. EMBO J.

[22] Braakhuis BJM, Tabor MP, Kummer JA, Leemans CR, Brakenhoff RH (2003). A genetic explanation of Slaughter’s concept of field cancerization: evidence and clinical implications. Cancer Res.

[23] Jaasma MJ, Jackson WM, Tang RY, Keaveny TM. Adaptation of cellular mechanical behavior to mechanical loading for osteoblastic cells. J Biomech. 40(9):1938–45. 10.1016/j.jbiomech.2006.09.010 17097091

[24] Orkin SH (1995). Transcription factors and hematopoietic development. J Biol Chem.

[25] Muntean AG, Pang L, Poncz M, Dowdy SF, Blobel GA, Crispino JD (2007). Cyclin D–Cdk4 is regulated by GATA-1 and required for megakaryocyte growth and polyploidization. Blood.

[26] Shivdasani RA, Orkin SH (1996). The transcriptional control of hematopoiesis. Blood.

[27] Krivtsov AV, Twomey D, Feng Z, Stubbs MC, Wang Y, Faber J, Levine JE, Wang J, Hahn WC, Gilliland DG (2006). Transformation from committed progenitor to leukaemia stem cell initiated by MLL-AF9. Nature.

[28] Chen Y, Hu Y, Zhang H, Peng C, Li S (2009). Loss of the Alox5 gene impairs leukemia stem cells and prevents chronic myeloid leukemia. Nat Genet.

[29] Rouault-Pierre K, Lopez-Onieva L, Foster K, Anjos-Afonso F, Lamrissi-Garcia I, Serrano-Sanchez M, Mitter R, Ivanovic Z, de Verneuil H, Gribben J (2013). HIF-2α protects human hematopoietic stem/progenitors and acute myeloid leukemic cells from apoptosis induced by endoplasmic reticulum stress. Cell Stem Cell.

[30] Shen H, Yu H, Liang PH, Cheng H, XuFeng R, Yuan Y, Zhang P, Smith CA, Cheng T (2012). An acute negative bystander effect of gamma-irradiated recipients on transplanted hematopoietic stem cells. Blood.

[31] Miraki-Moud F, Anjos-Afonso F, Hodby KA, Griessinger E, Rosignoli G, Lillington D, Jia L, Davies JK, Cavenagh J, Smith M (2013). Acute myeloid leukemia does not deplete normal hematopoietic stem cells but induces cytopenias by impeding their differentiation. Proc Natl Acad Sci U S A.

[32] Cheng T, Rodrigues N, Shen H, Yang Y-G, Dombkowski D, Sykes M, Scadden DT (2000). Hematopoietic stem cell quiescence maintained by p21cip1/waf1. Science.

[33] Cánepa ET, Scassa ME, Ceruti JM, Marazita MC, Carcagno AL, Sirkin PF, Ogara MF (2007). INK4 proteins, a family of mammalian CDK inhibitors with novel biological functions. IUBMB Life.

[34] Polager S, Ginsberg D (2009). p53 and E2f: partners in life and death. Nat Rev Cancer.

[35] Chang F, Lee JT, Navolanic PM, Steelman LS, Shelton JG, Blalock WL, Franklin RA, JA MC (2003). Involvement of PI3K/Akt pathway in cell cycle progression, apoptosis, and neoplastic transformation: a target for cancer chemotherapy. Leukemia.

[36] Chaussepied M, Ginsberg D (2004). Transcriptional regulation of AKT activation by E2F. Mol Cell.

[37] Wee KB, Aguda BD (2006). Akt versus p53 in a network of oncogenes and tumor suppressor genes regulating cell survival and death. Biophys J.

[38] Iwamoto K, Tashima Y, Hamada H, Eguchi Y, Okamoto M (2008). Mathematical modeling and sensitivity analysis of G1/S phase in the cell cycle including the DNA-damage signal transduction pathway. Biosystems.

[39] Shampine L, Reichelt M, Kierzenka J (1999). Solving index-1 DAEs in MATLAB and Simulink. SIAM Rev.

[40] Conn AR, Gould NIM, Toint PL: Trust-region methods. Philadelphia: Society for Industrial Mathematics; 1987.

[41] Kuznetsov YA (2004). Elements of applied bifurcation theory.

