# Supplementary figures and images for: System modeling reveals the molecular mechanisms of HSC cell cycle alteration mediated by Maff and Egr3 under leukemia
Source: BMC Syst Biol. 2017 Oct 3;11(Suppl 5):91. doi: 10.1186/s12918-017-0467-4 (PMC5629552; doi:10.1186/s12918-017-0467-4)

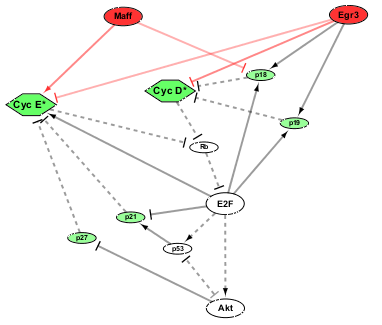

Supplement: Supplementary file 1 — Regulatory network with edge “Maff → Cdk2 (:Cyclin E)”. Another possible regulatory model includes an additional positive regulation on Cdk2/CyclinE by Maff, which is also capable of reproducing the qualitatively correct dynamic profiles illustrated by the experimental data (see Additional file 7: Table S1 for details). Here we do not discriminate the correctness between the model shown in Fig. 5 and the alternative one herein, as both models are qualitatively valid given the current data. The minimal model shown in Fig. 5 is chosen as example due to the principle of simplicity. (PNG 31 kb) [file 12918_2017_467_MOESM1_ESM.png]

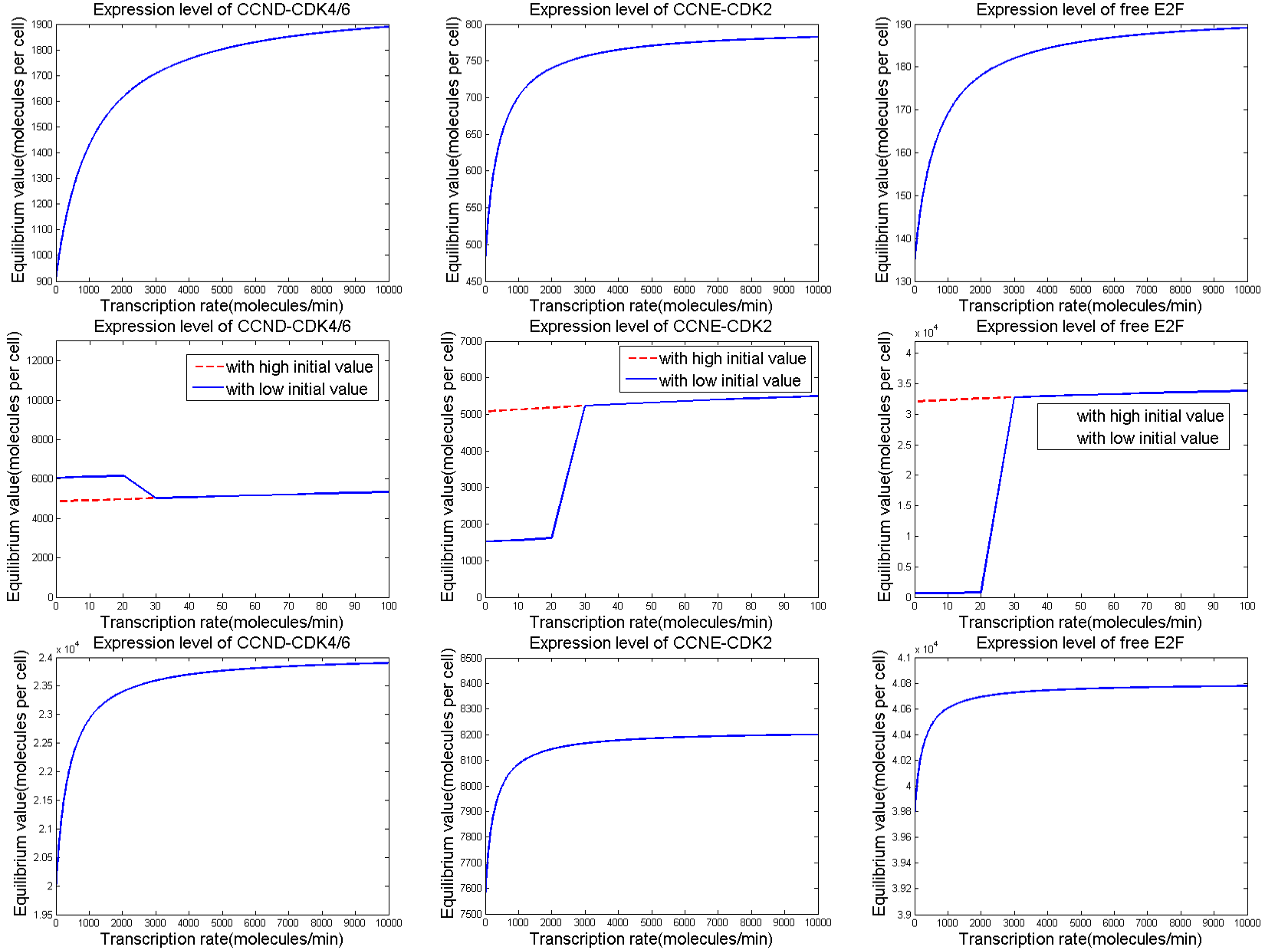

Supplement: Supplementary file 2 — Simulation results with additional regulation “Maff → Cdk2 (:Cyclin E)” with respect to Maff. Dynamics with respect to the transcription rate of Maff at high (upper), medium (middle), and low (lower) Egr3 expression-levels. In each panel, steady-state molecular quantities of Cyclin D-Cdk4/6 (left), Cyclin E-Cdk2 (middle) and E2F (right) are shown. The correct bistability with respect to Maff is qualitatively reproduced with the additional molecular action. (PNG 73 kb) [file 12918_2017_467_MOESM2_ESM.png]

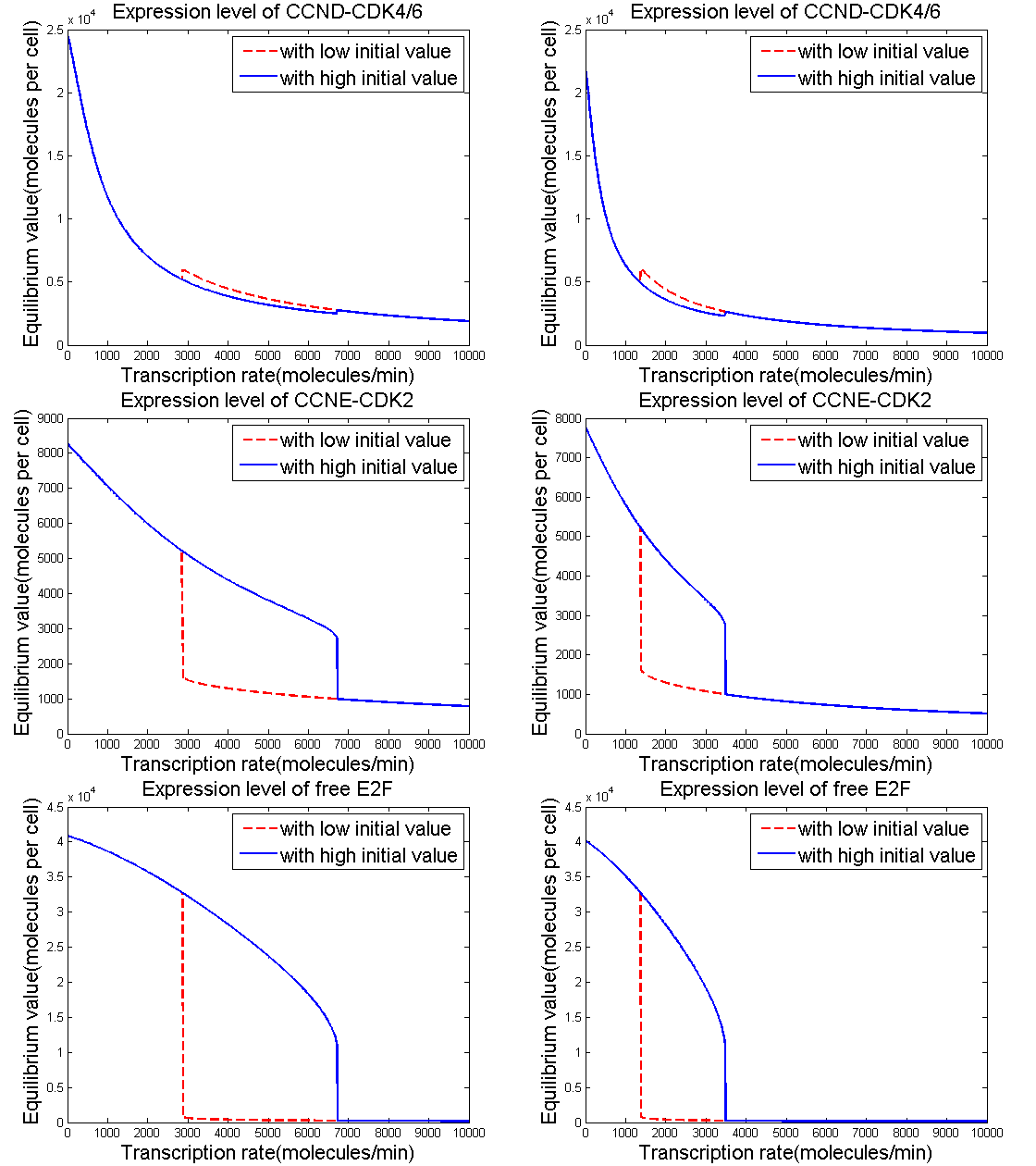

Supplement: Supplementary file 3 — Simualtion results with additional regulation “Maff → Cdk2 (:Cyclin E)” with respect to Egr3. Dynamics with respect to the transcription rate of Egr3 at high (upper) and low (lower) Maff expression-levels. In each panel, steady-state molecular quantities of Cyclin D-Cdk4/6 (left), Cyclin E-Cdk2 (middle) and E2F (right) are shown. The correct bistability with respect to Egr3 is qualitatively reproduced with the additional molecular action. (PNG 31 kb) [file 12918_2017_467_MOESM3_ESM.png]

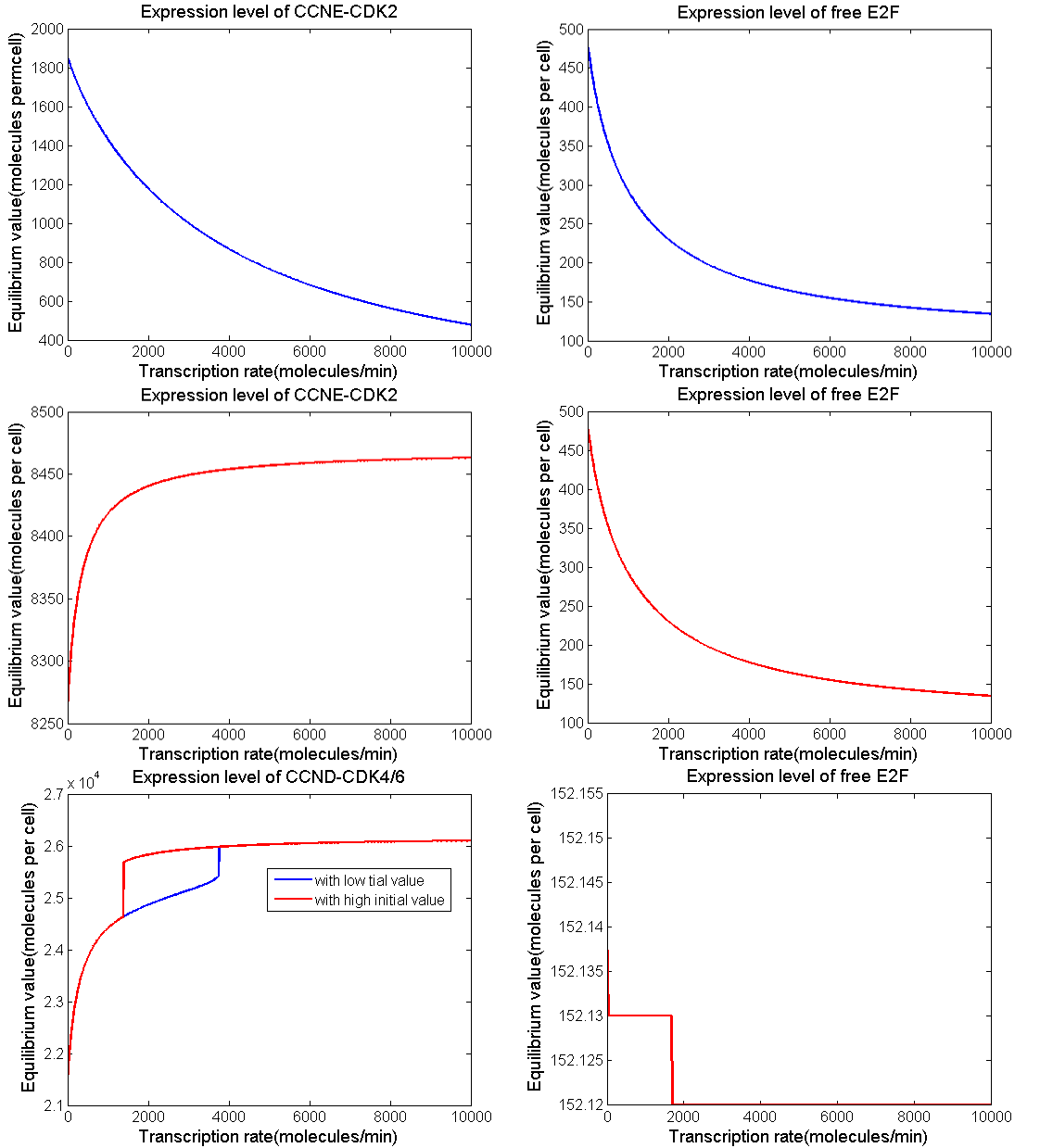

Supplement: Supplementary file 4 — Outcomes produced by other regulatory sturctures. Apparently false dynamics resulted by other hypotheses of regulations. Basically, all the other network structures than the one in Fig. 5/Additional file 1: Figure S1 produce qualitatively false results on (at least) one of Cdk4/6:CyclinD, Cdk2:CyclinE, and E2F. Here we show the most typically false results, combinations of regulatory relations are randomly assigned. Upper panel: unrealistic dynamic levels of Cdk2:CyclinE and E2F with respect to Maff transcription under low Egr3 expression, which is dictated by a randomly assigned network structure (regulatory code 1212); middle panel: results of Cdk2:CyclinE and E2F dictated by another network structure (regulatory code 2133); results of Cdk4/6:CyclinD and E2F dictated by a third different network structure (regulatory code 3321). Refer to Additional file 7: Table S1 for depiction of the regulatory codes. (PNG 52 kb) [file 12918_2017_467_MOESM4_ESM.png]

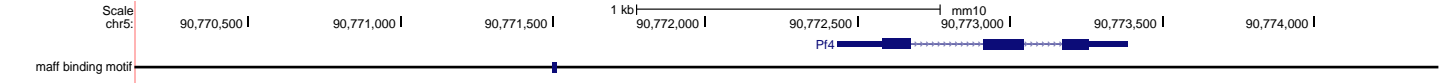

Supplement: Supplementary file 5 — Binding motif of Maff is also discovered within 2 kb upstream of Pf4 gene. The Maff binding motif for transcriptional activation occurs at a location <1 kb upstream the transcription start site (TSS) of Pf4, which is potentially within the promoter region of the gene. The observation indicated that Maff might positively regulate Pf4, which is a regulator of platelet formation. (PDF 5 kb) [file 12918_2017_467_MOESM5_ESM.pdf]
