# Supplementary material for: System modeling reveals the molecular mechanisms of HSC cell cycle alteration mediated by Maff and Egr3 under leukemia
Source: BMC Syst Biol. 2017 Oct 3;11(Suppl 5):91. doi: 10.1186/s12918-017-0467-4 (PMC5629552; doi:10.1186/s12918-017-0467-4)
Supplement: Supplementary file 6 — Formulations of the mathematical model. Descriptions for the ODEs and the modeling process are enclosed here. (DOC 128 kb) [file 12918_2017_467_MOESM6_ESM.doc]

**Description of ODE modeling**

The gene regulation network of G0/G1/S phase-transition controls the initiation of cell cycle, determines patterns of cell division, and also couples with cell apoptosis and survival . The expression of CyclinD rises at early stage of G1 phase and stablizes during the cycle. CyclinD binds to Cdk4 or Cdk6 (Cdk4/6) to be activated; and the active complex CyclinD(:Cdk4/6) partially phosphorylates and inhibits the activity of Rb, which binds to E2F and inhibits its activity. The CKIs in G1 phase, p18ink4c (p18) and p19ink4d (p19), bind to CDK4/6 and inhibit (the active) CyclinD . E2F is the most important TF in G0/G1/S phases that (up-)regulates many target genes, including p18, p19, Cyclin E, etc. . After being (partially) released in the midst of phase G1, free E2F upregulates the expression of CyclinE. Moreover, CyclinE binds to Cdk2 to totally phosphorylate and inhibit Rb; and thus free E2F can be completely released. The S-phase CKIs, p21 and p27, bind to Cdk2 and inhibit CyclinE. In short, CyclinD, CyclinE and E2F constitute a positive feedback loop in the G0/G1/S network. Generally, expression levels of CyclinE and E2F reach the maximum in the S-phase checkpoint to impel the cell into the following G2/M phases. The G0/G1/S network also interacts with the networks of cell apoptosis and survival. p53 is the most important TF in (the upstream of) the apoptosis network, which is upregulated by E2F indirectly via a series of pathways. And p53 in turn suppresses E2F by the p21 and mir-34a pathways . AKT, core of the survival network, is positively regulated by E2F. It upregulates E2F via inhibiting p21 and p27 or promoting CyclinD . In addition, AKT and p53 inhibit each other to form a negative feedback .

Transcription regulation is described by first-order Hill function of transcription factors. For example, the maim part of transcription rate of CCNE regulated by E2F is described as :

$$ks\_E2F\_CCNE*\frac{|E2F|}{M+|E2F|}$$

Where |E2F| is expression level of E2F.

Funcion of negative transcription regulation such as maff inhibiting expression level of p18is described as:

$$\frac{|maff|}{M+|maff|}$$

Degradation rates are assumed linear relationship. Moreover, when degradation rate is promoted by other molecule, first-order Hill function is used to describe the relationship such as p19 promoting degradation of CDK46 as follows:

$$kd\_p19\_CDK46*|p19|*\frac{|CCNDCDK46|}{M+|CCNDCDK46|}$$

the same as regulation of activation or inactivation, too.

Binding rate between is assumed as linear relationship such as binding betweer CDK2 and CCNE as follows:

$$kb*|CCNE|*|CDK2|$$

and unbinding rate of its reverse reaction is assumed as linear relationship.

$\begin{matrix} \frac{dCCND}{dt}=ks\_CCND-kb*|CCND|*|CDK46|+kub*|CCNDCDK46| \\ -(kd-kud\_AKT\_CCND*\frac{AKTa}{M+AKTa})*|CCND| \end{matrix}$...[1]

$\begin{matrix} \frac{dCDK46}{dt}=ks\_CDK46*\frac{M}{M+|Egr3|}-kb*|CCND|*|CDK46|+kub*|CCNDCDK46|-kd*|CDK46| \\ -kd\_p18\_CDK46*|p18|*\frac{|CCNDCDK46|}{M+|CCNDCDK46|}-kd\_p19\_CDK46*|p19|*\frac{|CCNDCDK46|}{M+|CCNDCDK46|} \end{matrix}$...[2]

$\begin{matrix} \frac{dp18}{dt}=(ks\_p18+ks\_E2F\_p18*\frac{|E2F|}{M+|E2F|}+ks\_Egr3\_p18*\frac{|Egr3|}{M+|Egr3|}) \\ *\frac{M}{M+|maff|}-kd*|p18| \end{matrix}$...[3]

$\frac{dp19}{dt}=(ks\_p19+ks\_E2F\_p19*\frac{|E2F|}{M+|E2F|}+ks\_Egr3\_p19*\frac{|Egr3|}{M+|Egr3|})-kd\_p19*|p19|$...[4]

$\begin{matrix} \frac{dCCNDCDK46}{dt}=kb*|CCND|*|CDK46|+kub*|CCNDCDK46| \\ -kd\_CCNDCDK46*|CCNDCDK46| \end{matrix}$...[5]

$\frac{dRb}{dt}=-kp\_CCNDCDK46*\frac{|Rb|}{M+|Rb|}+kdp\_Rdp*|Rdp|$...[6]

$\begin{matrix} \frac{dRdp}{dt}=kp\_CCNDCDK46*\frac{|Rb|}{M+|Rb|}-kdp\_Rdp*|Rdp| \\ -kp\_CCNECDK2*|CCNECDK2|*\frac{|Rdp|}{M+|Rdp|}+kdp\_Rdpp*|Rdpp| \end{matrix}$...[7]

$\frac{dRbpp}{dt}=kp\_CCNECDK2*|CCNECDK2|*\frac{|Rdp|}{M+|Rdp|}-kdp\_Rdpp*|Rdpp|$...[8]

$\begin{matrix} \frac{dE2F}{dt}=ks\_E2F+ks\_E2F\_E2F*\frac{|E2F|}{M+|E2F|} \\ -kd\_E2F*|E2F|-kd\_Rb\_E2F*(|Rb|+|Rbp|)*\frac{|E2F|}{M+|E2F|} \end{matrix}$...[9]

$\begin{matrix} \frac{dCCNE}{dt}=ks\_CCNE+ks\_E2F\_CCNE*\frac{|E2F|}{M+|E2F|}-kd*|CCNE| \\ -kb*|CCNE|*|CDK2|+kub*|CCNECDK2| \end{matrix}$...[10]

$\begin{matrix} \frac{dCDK2}{dt}=ks\_CDK2*\frac{M}{M+|Egr3|}-kd*|CDK2| \\ -kb*|CCNE|*|CDK2|+kub*|CCNECDK2| \end{matrix}$...[11]

$\begin{matrix} \frac{dp21}{dt}=ks\_p21+ks\_p53\_p21*\frac{|p53|}{M+|p53|+ki\_E2F\_p53*|E2F|} \\ -(kd\_p21-kud\_AKT\_p21*\frac{|AKTa|}{M+|AKTa|}) \\ -kb*|p21|*|CCNECDK2|*\frac{M}{M+|AKTa|}\_kub*|CCNECDK2p21| \end{matrix}$...[12]

$$\begin{matrix} \frac{dp27}{dt}=ks\_p27*\frac{M}{M+|AKTa|}-kd*|p27| \\ -kb*|p27|*|CCNECDK2|*\frac{M}{M+|AKTa|}+kub*|CCNECDK2p27| \\ -kd\_AKT\_p27*|AKTa|*\frac{|p27|}{M+|p27|}-kd\_CCNECDK2\_p27*|CCNECDK2|*\frac{|p27|}{M+|p27|} \end{matrix}$$

...[13]

$\begin{matrix} \frac{dCCNECDK2}{dt}=kb*|CCNE|*|CDK2|-kub*|CCNECDK2|-kd*|CCNECDK2| \\ -kb*|p21|*|CCNECDK2|*\frac{M}{M+|AKTa|}+kub*|CCNECDK2p21| \\ -kb*|p27|*|CCNECDK2|*\frac{M}{M+|AKTa|}+kub*|CCNECDK2p27| \end{matrix}$.

..[14]

$\begin{matrix} \frac{dCCNECDK2p21}{dt}=kb*|p21|*|CCNECDK2|*\frac{M}{M+|AKTa|} \\ -kub*|CCNECDK2p21|-kd*|CCNECDK2p21| \end{matrix}$...[15]

$\begin{matrix} \frac{dCCNECDK2p27}{dt}=kb*|p27|*|CCNECDK2|*\frac{M}{M+|AKTa|} \\ -kub*|CCNECDK2p27|-kd*|CCNECDK2p27| \end{matrix}$...[16]

$\begin{matrix} \frac{dp53}{dt}=ks\_p53+ks\_p53\_p53*\frac{|p53|}{M+|p53|} \\ \frac{kd*|p53|+kd\_AKT\_p53*|AKTa|*\frac{|p53|}{M+|p53|+|E2F|}}{1+kud\_E2F\_p53*\frac{|E2F|}{M+|AKTa|}} \end{matrix}$...[17]

$\begin{matrix} \frac{dAKTa}{dt}=ka\_AKT*|AKTi|+ka\_E2F\_AKT*|E2F|*\frac{|AKTi|}{M+|AKTi|} \\ -ki\_AKT*|AKTa|-ki\_p53\_AKT*|p53|*\frac{|AKTa|}{M+|AKTa|} \end{matrix}$...[18]

$\frac{dmaff}{dt}=ks\_maff-kd*|maff|$...[19]

$\frac{dEgr3}{dt}=ks\_Egr3-kd*|Egr3|$...[20]

**References cited**

1. Qu Z, Weiss JN, MacLellan WR: **Regulation of the mammalian cell cycle: a model of the G1-to-S transition**. *Am J Physiol Cell Physiol* 2003, **284**(2):C349-C364.

2. Cánepa ET, Scassa ME, Ceruti JM, Marazita MC, Carcagno AL, Sirkin PF, Ogara MF: **INK4 proteins, a family of mammalian CDK inhibitors with novel biological functions**. *IUBMB Life* 2007 **59**(7):419-426.

3. Haberichter T, Mädge B, Christopher RA, Yoshioka N, Dhiman A, Miller R, Gendelman R, Aksenov SV, Khalil IG, Dowdy SF: **A systems biology dynamical model of mammalian G1 cell cycle progression**. *Mol Syst Biol* 2007, **3**(1).

4. Polager S, Ginsberg D: **p53 and E2f: partners in life and death**. *Nat Rev Cancer* 2009, **9**(10):738-748.

5. Chang F, Lee JT, Navolanic PM, Steelman LS, Shelton JG, Blalock WL, Franklin1 RA, McCubrey JA: **Involvement of PI3K/Akt pathway in cell cycle progression, apoptosis, and neoplastic transformation: a target for cancer chemotherapy**. *Leukemia* 2003, **17**:590–603.

6. Chaussepied M, Ginsberg D: **Transcriptional regulation of AKT activation by E2F**. *Mol Cell* 2004, **16**:831–837.

7. Wee KB, Aguda BD: **Akt versus p53 in a network of oncogenes and tumor suppressor genes regulating cell survival and death**. *Biophys J* 2006 **91** 857–865.
